# The descriptive epidemiology of pre-omicron SARS-CoV-2 breakthrough infections and severe outcomes in Manitoba, Canada

**DOI:** 10.3389/fepid.2023.1248847

**Published:** 2024-01-12

**Authors:** Souradet Y. Shaw, Jason Kindrachuk, Lyle McKinnon, Jeffery C. S. Biegun, Jocelyn N. Reimer, Carla Loeppky, Yichun Joy Wei, Jared Bullard, Paul Van Caeseele, Derek R. Stein

**Affiliations:** ^1^ Department of Community Health Sciences, University of Manitoba, Winnipeg, MB, Canada; ^2^ Department of Medical Microbiology and Infectious Diseases, University of Manitoba, Winnipeg, MB, Canada; ^3^ Department of Sociology and Criminology, University of Manitoba, Winnipeg, MB, Canada; ^4^ Winnipeg Regional Health Authority, Winnipeg, MB, Canada; ^5^ Manitoba Health, Winnipeg, MB, Canada; ^6^ Cadham Provincial Laboratory, Winnipeg, MB, Canada

**Keywords:** COVID-19, breakthrough infection, severe outcomes, chronic conditions, vaccination

## Abstract

**Introduction:**

Vaccination plays a key role in curbing severe outcomes resulting from COVID-19 disease. With the Omicron variant and the relaxing of public health protections breakthrough infections are increasingly common, and certain groups remain at higher risk for severe outcomes from breakthrough infections. We analysed population-based public health data from Manitoba, Canada to understand characteristics of those experiencing breakthrough infections and severe outcomes from breakthrough infections. Data from previous pandemic stages can provide valuable information regarding severe outcomes associated with breakthrough infection in the Omicron and future phases.

**Methods:**

Positive SARS-CoV-2 PCR tests from Cadham Provincial Laboratory were linked to case information from the population-based Public Health Information Management System. A retrospective design was used with time-to-event analyses to examine severe outcomes among those experiencing breakthrough infection.

**Results:**

Breakthrough cases were more likely to have 2 + chronic conditions, compared to age-, sex-, and time-period matched unvaccinated cases (24% vs. 17%), with hypertension (30%), diabetes (17%), and asthma (14%) being the most prevalent chronic conditions amongst breakthrough cases. Severe outcomes resulting from breakthrough infection was associated with age and chronic conditions, with those with 2 + chronic conditions at higher risk of severe outcomes (adjusted hazard ratio: 3.6, 95% confidence intervals: 2.0-6.4). Risk of severe outcomes varied by age group, with those 70 + years at over 13 times the risk of severe outcomes (95% CI: 4.5-39.8), compared to those 18-29 years of age.

**Discussion:**

Our results demonstrate the impact of chronic conditions on the likelihood of, and severity of outcomes from breakthrough infections. These findings underscore the importance of vaccination programs prioritizing vulnerable populations.

## Introduction

The speed and scale at which SARS-CoV-2, the virus that causes COVID-19 disease, has impacted citizens globally has been staggering (1), with over half a billion reported infections, and deaths numbering in the millions as of July 2022 (2). Although COVID-19 vaccines have successfully curbed COVID-19 morbidity and mortality, vaccines by themselves have proven to be *necessary*, but *insufficient* to eliminate the deleterious impacts of COVID-19 (3). This is especially true in light of waning vaccine effectiveness (4), and the challenges associated with the immune-evading Omicron variant (5, 6, 7). Omicron has rendered breakthrough infections (i.e., infections that occur after a full course of COVID-19 vaccinations) the rule, rather than the exception (7, 8). At the same time, evidence remains of the effectiveness of booster doses in mitigating severe outcomes associated with COVID-19 (9). As most public health protections have been lifted across Canada, the main public policy approach has been through continued vaccination coverage and provision of boosters to targeted segments of the population, with eligibility criteria and uptake heterogeneous across jurisdictions. Moreover, with changing epidemiology and approaches to testing, public health authorities across Canada have been challenged to diagnose and track SARS-CoV-2 infections, limiting knowledge about those most susceptible to breakthrough infections. Although breakthrough infections are associated with reduced risk of severe outcomes, including hospitalization and death (10, 11), a significant proportion of breakthrough cases experience severe outcomes (7, 12–15), necessitating a need to identify those at risk for severe outcomes. In most jurisdictions, populations who are most vulnerable to severe outcomes from COVID-19 have been prioritized in vaccination campaigns, including seniors, and those with comorbid conditions. An important consideration for healthcare planning are understanding the characteristics of vaccinated individuals who experience breakthrough infections. Identification of high-risk populations for breakthrough infections could inform earlier mitigation strategies, such as targeting of public health recommendations, boosters, and therapeutics (12). Thus, although the world is in the Omicron phase of the pandemic, there is much to learn from previous waves.

The province of Manitoba has been one of the hardest struck Canadian provinces (16). At the end of July 2022, Manitoba's cumulative mortality rate, at 149/100,000 population, was second only to the province of Quebec (185/100,000 population) (17), although heterogeneity in how COVID-19 mortality is captured may be contributing to observed regional differences (18). Understanding who is most at risk for breakthrough infections, and how they differ from those who are unvaccinated, as well as characterizing those breakthrough cases most impacted by severe outcomes, can help guide prevention policy, especially in light of more nuanced public health responses being needed as the pandemic enters its third year (19). The main objective of this study was to describe the characteristics of those experiencing pre-Omicron SARS-CoV-2 breakthrough infections, with an emphasis on the association of chronic conditions and breakthrough infections. A secondary objective was to examine the correlates of hospitalizations and ICU admissions among a cohort of Manitobans experiencing breakthrough infections during this period.

## Materials and methods

### Setting & data sources

In 2021, Manitoba had a population of 1.4 million, with approximately 60% (*n* = 791,284) of Manitoba's population residing in the city of Winnipeg. Demographic and clinical information from case and contact investigations on all diagnosed COVID-19 cases in Manitoba are maintained in the provincial Public Health Information Management System (PHIMS). Prior to the Omicron wave in December 2022, trained public health nurses were responsible for investigations of all confirmed COVID-19 cases in Manitoba. Vaccination information on all Manitobans is also maintained in PHIMS. Positive SARS-CoV-2 PCR test results from Cadham Provincial Laboratory (CPL) were linked to case information through personal health identification numbers. Prevalent chronic conditions were determined through validated algorithms of the Canadian Chronic Disease Surveillance System (CCDSS), using administrative health records maintained by Manitoba Health, and data linkage performed by Manitoba Health analysts (20). Hospitalizations, ICU admissions, and deaths (including dates) were defined through case investigations. For the purposes of these analyses only information on PCR-positive cases was available.

### Analyses

A retrospective cohort design was used for analyses. Cases were considered fully vaccinated if their records indicated having at least two vaccination doses. Epidemiological date was defined as the earliest date of symptom onset or specimen collection date of the laboratory test. Breakthrough infections were defined as fully vaccinated individuals who had a PCR-positive test result >14 days (as measured by epi-date) after their last vaccination dose. Any cases with a COVID-19 infection prior to the study period (i.e., before January 1, 2021) were excluded, as were any cases under the age of 18 at epi-date. Only cases who had an epi-date prior to December 1st, 2021 were included, to address the arrival of the Omicron variant in Manitoba. In addition to summary statistics of the characteristics of COVID-19 cases who had breakthrough infections, we matched breakthrough cases to contemporaneous unvaccinated cases (i.e., cases with no history of vaccination recorded), based on epi-date (within +/− 7 days of the breakthrough case's epi-date), age group, and sex, at a 1:4 ratio. Odds ratios and their 95% confidence intervals (95% CI) from conditional logistic regression models are reported. Chronic conditions captured in the CCDSS included asthma, chronic obstructive pulmonary disease (COPD), diabetes, epilepsy, heart failure, hypertension, ischemic heart disease (IHD), multiple sclerosis, myocardial infarction, osteoarthritis, Parkinson's disease (PD), and stroke.

For the second objective, time-to-event analyses were used to examine the correlates of severe outcomes from breakthrough infections. All reported breakthrough infection cases in Manitoba with an epi-date between January 1 and November 30, 2021 were included. The outcome measure was defined as any evidence of hospitalization or ICU admission up to December 31, 2021, which was determined through case investigation by public health nurses. Only the date of first admission was recorded; subsequent readmissions were not captured, and thus, individuals could only appear once in the analytical databases. Mortality was not examined separately, as mortality outside of hospital settings was not included in our dataset. Epi-date of infection and admission date was used to record time between events. To address nosocomial COVID-19 infections, all individuals with an epi-date after their hospital admission were excluded, as were individuals who were admitted the same day of their epi-date. Sex, age group, dose interval (days between 1st and 2nd doses), RHA, and number of chronic conditions were included in bivariate and multivariable Cox regression models. Crude and adjusted hazard ratios (AHR) and their 95% CI are reported. Proportional hazards assumptions were tested using log-log plots and tests of Schoenfeld residuals. No violation of the proportional hazards assumption was detected. Lastly, the weekly incidence of breakthrough infections was plotted against all other incident infections during the study period. For all analyses, individuals who only had the Johnson & Johnson vaccine (*n* = 89) were excluded from the analyses. All analyses were performed at CPL using Stata V17 (College Station, TX). Ethics approval was waived by the Human Research Ethics Board at the University of Manitoba, as this was a secondary analysis of routinely-collected public health data.

## Results

**Figure 1:**
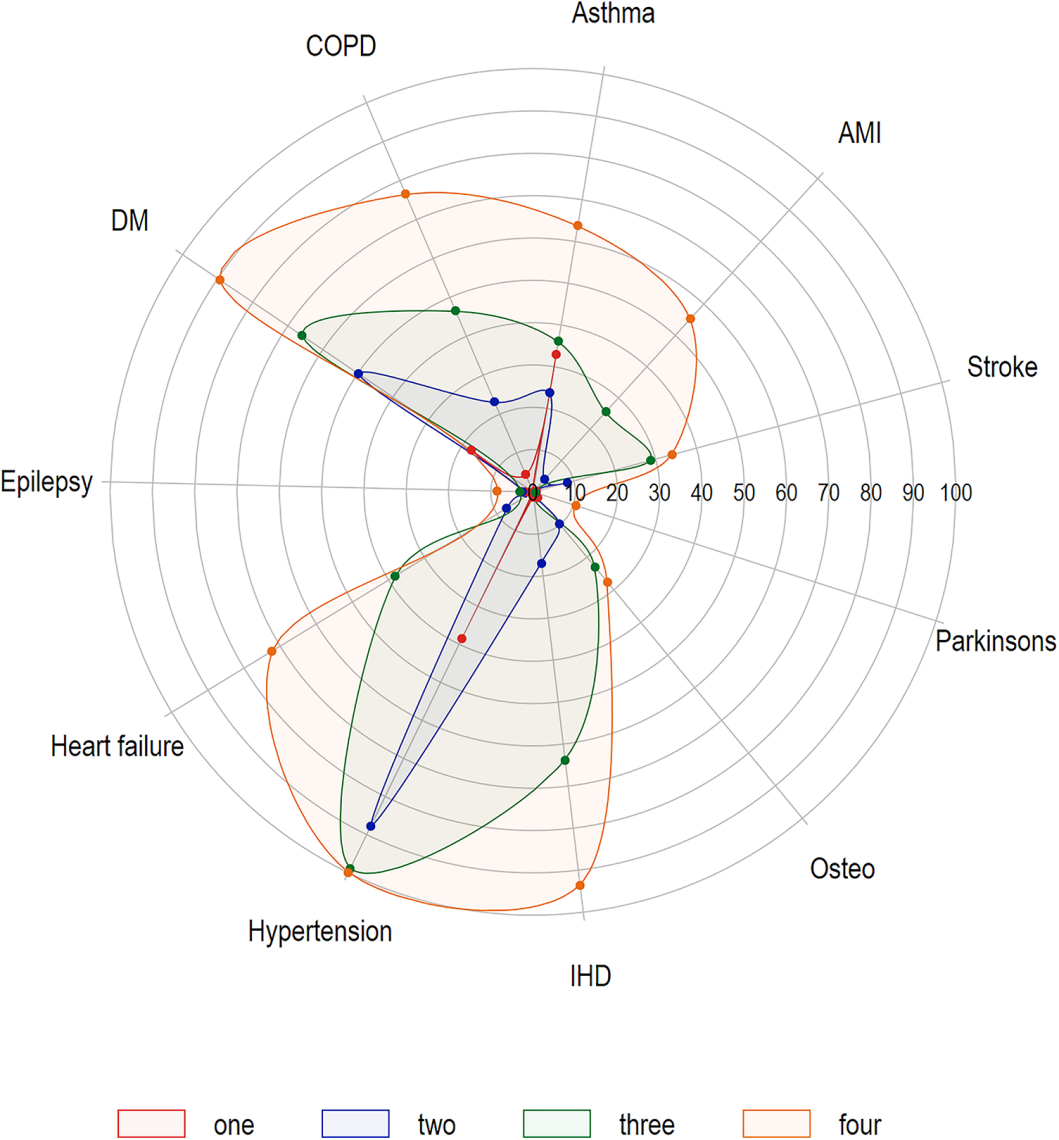
Spider plot of chronic conditions prevalence (%), by number of chronic conditions (one, two, three, four plus), fully-vaccinated adults (18 + years) experiencing SARS-CoV-2 breakthrough infections in Manitoba, January 1–November 30, 2021 (*N* = 3,803)*.

A total of 3,807 breakthrough infections in adults 18 years and older were reported between January 1st, 2021 and November 30, 2021. Of these, four were excluded because their epi-dates occurred before their first vaccine dose date. Thus, 3,803 individuals with breakthrough infections were included (Table 1). Approximately 55% of reported breakthrough infections were females, and 66% occurring in those <55 years of age; of note, 47% of breakthrough infections occurred in the month of November alone. Compared to age group, sex, and time-matched unvaccinated cases, breakthrough cases were more likely to have 2 + chronic conditions (24% vs. 17%; OR: 2.0, 95% CI: 1.7–2.2). Hypertension, diabetes, and asthma, at 30%, 17%, and 14%, respectively, were the most common chronic conditions reported. Compared to contemporaneous age- and sex-matched unvaccinated cases, breakthrough cases were at 1.5 (95% CI: 1.3–1.6), 1.7 (95% CI: 1.6–1.9), and 1.2 (95% CI: 1.1–1.3) times the odds of having a diagnosis of hypertension, diabetes, and asthma, respectively. Figure 1 is a spider plot showing the prevalence of chronic conditions by the number of chronic conditions (1, 2, 3, and 4+) diagnosed in adult breakthrough cases. Regardless of how many chronic conditions were diagnosed, hypertension was the most prevalent condition reported. Of those with four or more chronic conditions, hypertension was present in 100% of breakthrough cases, IHD in 92%, and diabetes in 90%. The median time from the second dose to breakthrough infection was 131, with an IQR of 94–163 days. Supplementary Figure S1 contains a histogram of the days to infection for those who were fully vaccinated, diagnosed prior to the Omicron period, and were a breakthrough case.

**Table 1:** Selected characteristics, breakthrough infections in fully vaccinated cases compared to contemporaneous matched unvaccinated cases in Manitoba (January 1–November 30, 2021), and odds ratios (ORs) and 95% confidence intervals (95% CI) from conditional logistic regression models (*N* = 15,216)^a^.

	Unvaccinated cases		Breakthrough cases		Total		ORs (95% CI)
	No.	%	No.	%	No.	%	
Sex^a^							–
Female	6,267	54.9	2,089	54.9	8,356	54.9	
Male	5,145	45.1	1,715	45.1	6,860	45.1	
Age group							
18–29	1,920	16.8	640	16.8	2,560	16.8	*–*
30–39	2,304	20.2	768	20.2	3,072	20.2	
40–49	2,208	19.3	736	19.3	2,944	19.3	
50–59	1,674	14.7	558	14.7	2,232	14.7	
60–69	1,494	13.1	498	13.1	1,992	13.1	
70+	1,812	15.9	604	15.9	2,416	15.9	
Regional health authority							
Interlake-Eastern	886	7.8	338	8.9	1,224	8.0	0.56 (0.48–0.66)
Northern	588	5.2	620	16.3	1,208	7.9	1.60 (1.39–1.84)
Prairie Mountain	1,313	11.5	504	13.2	1,817	11.9	0.49 (0.43–0.56)
Southern	6,404	56.1	923	24.3	7,327	48.2	0.19 (0.17–0.21)
Winnipeg	2,221	19.5	1,419	37.3	3,640	23.9	Ref
Month (epi-date)^a^							–
Feb	2	0.0	1	0.0	3	0.0	
Mar	32	0.3	9	0.2	41	0.3	
Apr	96	0.8	31	0.8	127	0.8	
May	390	3.4	127	3.3	517	3.4	
Jun	206	1.8	64	1.7	270	1.8	
Jul	308	2.7	99	2.6	407	2.7	
Aug	752	6.6	277	7.3	1,029	6.8	
Sep	1,636	14.3	518	13.6	2,154	14.2	
Oct	2,957	25.9	917	24.1	3,874	25.5	
Nov	5,033	44.1	1,761	46.3	6,794	44.7	
Chronic conditions							
0	7,060	61.9	2,039	53.6	9,099	59.8	Ref
1	2,364	20.7	862	22.7	3,226	21.2	1.37 (1.24–1.51)
2	1,499	13.1	665	17.5	2,164	14.2	1.89 (1.67–2.13)
3	372	3.3	191	5.0	563	3.7	2.37 (1.94–2.90)
4+	117	1.0	47	1.2	164	1.1	1.94 (1.36–2.77)
Stroke							
No	11,055	96.9	3,670	96.5	14,725	96.8	Ref
Yes	357	3.1	134	3.5	491	3.2	1.14 (0.92–1.40)
Heart failure							
No	11,124	97.5	3,643	95.8	14,767	97.0	Ref
Yes	288	2.5	161	4.2	449	3.0	1.81 (1.47–2.23)
Acute myocardial infarction							
No	11,152	97.7	3,702	97.3	14,854	97.6	Ref
Yes	260	2.3	102	2.7	362	2.4	1.19 (0.94–1.52)
Ischemic heart disease							
No	11,152	97.7	3,702	97.3	14,854	97.6	Ref
Yes	260	2.3	102	2.7	362	2.4	1.49 (1.28–1.75)
Diabetes							
No	10,125	88.7	3,153	82.9	13,278	87.3	Ref
Yes	1,287	11.3	651	17.1	1,938	12.7	1.73 (1.55–1.93)
Hypertension							
No	8,540	74.8	2,652	69.7	11,192	73.6	Ref
Yes	2,872	25.2	1,152	30.3	4,024	26.4	1.47 (1.33–1.63)
Chronic obstructive pulmonary disease							
No	10,682	93.6	3,486	91.6	14,168	93.1	Ref
Yes	730	6.4	318	8.4	1,048	6.9	1.40 (1.20–1.62)
Asthma							
No	10,007	87.7	3,263	85.8	13,270	87.2	Ref
Yes	1,405	12.3	541	14.2	1,946	12.8	1.18 (1.06–1.32)
Parkinson's							
No	11,388	99.8	3,793	99.7	15,181	99.8	Ref
Yes	24	0.2	11	0.3	35	0.2	1.38 (0.67–2.81)
Multiple sclerosis							
No	11,382	99.7	3,798	99.8	15,180	99.8	Ref
Yes	30	0.3	6	0.2	36	0.2	0.60 (0.25–1.44)
Epilepsy							
No	11,355	99.5	3,761	98.9	15,116	99.3	Ref
Yes	57	0.5	43	1.1	100	0.7	2.29 (1.54–3.42)
Osteoarthritis							
No	11,188	98.0	3,667	96.4	14,855	97.6	Ref
Yes	224	2.0	137	3.6	361	2.4	2.13 (1.67–2.72)

^a^ Matched on age group, sex, and epidemiological date (1:4 ratio).

For Aim 2, and from 3,803 individuals with breakthrough infections, 6.9% experienced a severe outcome (*n* = 264). Of these 264 individuals, 98 had an epi-date after their hospital admission. These 98 individuals were excluded from analyses, leaving a total of 166 individuals who experienced a severe outcome. Thus, 3,706 individuals were retained in Cox regression models examining correlates of severe outcomes (Table 2). Of these, median time of severe outcomes was 3.5 days (IQR: 1–8 days), while the median age of the breakthrough infection cohort was 45 years (IQR: 33–61), with those experiencing severe outcomes (median: 70, IQR: 59–83) older than those who did not (median: 44, IQR: 33–60). Correspondingly, 52% of the severe outcome group were 70 years or older, compared to 13% of those not experiencing severe outcomes. In fully-adjusted models, no violation of the proportional hazards assumption was detected. Fully-adjusted models did not detect any statistically significant differences due to interval dose, and showed those with 2 + chronic conditions were more likely to experience severe outcomes, compared to those with no recorded chronic condition (AHR: 3.6, 95% CI: 2.0–6.4). Age group was significantly associated with the risk of severe outcomes; relative to those 18–29 years of age, risk for severe outcomes was almost 6-fold higher for those 60–69 years of age (AHR: 5.8, 95% CI: 1.9–17.6), and over 13-fold (95% CI: 4.5–39.8) for those 70 + years of age. Table 3 shows cause-specific AHRs for each chronic condition and the risk of severe outcomes; those with a diagnosis of heart failure (AHR: 3.3, 95% CI: 2.2–4.9) and hypertension (AHR: 2.4, 95% CI: 1.5–3.8) were at the highest risk for severe outcomes.

**Table 2:** Unadjusted and adjusted hazard ratios (U/AHRs) and 95% confidence intervals (95% CI) from Cox regression models examining determinants of hospitalizations/ICU admissions amongst breakthrough infection cases in Manitoba, January 1–November 30, 2021 (*N* = 3,706).

Variables		Hospitalized/ICU			
		No No. (%)	Yes No. (%)	AHR	95% CI
		3,540 (96.2)	166 (4.5)	** **	** **
Sex	Female	1,945 (54.9)	83 (50.0)	Ref	–
	Male	1,595 (45.1)	83 (50.0)	1.26	[0.90, 1.77]
Age group	18–29	630 (17.8)	6 (3.6)	Ref	–
	30–39	757 (21.4)	8 (4.8)	1.04	[0.28, 3.87]
	40–49	718 (20.3)	15 (9.0)	2.12	[0.67, 6.70]
	50–59	533 (15.1)	14 (8.4)	1.93	[0.58, 6.41]
	60–69	445 (12.6)	37 (22.3)	5.80*	[1.91, 17.59]
	70+	457 (12.9)	86 (51.8)	13.3**	[4.45, 39.76]
	Mean (years), Median (IQR)	47.1, 44 (33–60)	71.0, 70 (59–83)		
1st & 2nd dose	<30 days	935 (26.4)	45 (27.1)	Ref	–
interval	30–59 days	1,797 (50.8)	78 (47.0)	1.33	[0.86, 2.06]
	60 + days	808 (22.8)	43 (25.9)	0.68	[0.42, 1.10]
Regional health authority	Interlake-Eastern	312 (8.8)	16 (9.6)	0.57	[0.31, 1.02]
	Northern	588 (16.6)	24 (14.5)	0.71	[0.41, 1.22]
	Prairie Mountain	451 (12.7)	27 (16.3)	0.73	[0.44, 1.23]
	Southern	862 (24.4)	34 (20.5)	0.45*	[0.27, 0.74]
	Winnipeg	1,327 (37.5)	65 (39.2)	Ref	–
Chronic conditions	0	2,001 (56.5)	24 (14.5)	Ref	–
	1	817 (23.1)	31 (18.7)	1.57	[0.83, 2.97]
	2+	722 (20.4)	111 (66.9)	3.55**	[1.98, 6.38]
	Median (IQR)	0 (0–1)	2 (1–4)		

* *p* < 0.01.

** *p* < 0.001.

**Table 3:** Adjusted hazard ratios (AHRs^a^) and 95% confidence intervals (95% CI) from separate Cox regression models, association between specific chronic conditions and hospitalizations/ICU admissions among breakthrough infection cases in Manitoba, January 1–November 30, 2021 (*N* = 3,706).

		Prevalence (%)	
Condition	AHR (95% CI)^a^	Non- hospitalized	Hospitalized
Stroke	1.19 (0.69–2.08)	3.0	11.2
Heart failure	**3.25** **(2.15–4.93)**	2.89	26.9
Myocardial infarction	1.66 (0.94–2.94)	2.2	10.4
Ischemic heart disease	**1.63** (**1.09–2.44)**	6.2	28.4
Diabetes	**1.99** (**1.38–2.87)**	15.0	44.0
Hypertension	**2.41** (**1.51–3.84)**	27.0	74.6
COPD	**1.74** (**1.17–2.58)**	6.9	29.1
Asthma	1.35 (0.87–2.11)	14.0	17.9
Parkinson's disease	2.41 (0.59–9.88)	0.2	1.5
Multiple sclerosis	–	–	–
Epilepsy	1.36 (0.43–4.28)	1.0	2.2
Osteoarthritis	1.58 (0.94–2.66)	2.8	14.9
Any chronic condition	**2.81** (**1.55–5.08)**	45.8	88.1

^a^ Adjusted for age group, sex, dose interval, & regional health authority.

Bold values statistically significant at *p*<0.05 level.

**Figure 2:**
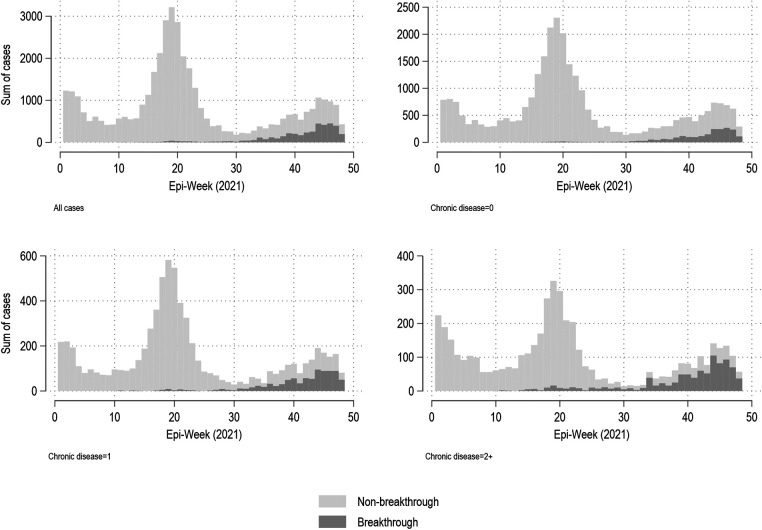
Weekly summary of all reported COVID-19 cases, by breakthrough and chronic disease status, Manitoba (January 1, 2021–November 30, 2021)*. *Note different y-axes based on chronic disease status.

Figure 2 shows the weekly summary of reported COVID-19 cases in Manitoba from January 1 to November 30, 2021, based on epi-date. Figures are stratified by number of chronic conditions, and by breakthrough status. Although the majority of infections were from those with no recorded chronic diseases, breakthrough infections were associated with chronic disease status, being especially prevalent in those with 2 + chronic conditions. For example (data not shown), in weeks 45–48, there were a total of 3,316 reported COVID-19 infections among those with no chronic conditions recorded, of which 44% (*n* = 1,458) were breakthrough infections. During this same time period, there were 567 infections among those with one recorded chronic condition, of which 56% (*n* = 317) were breakthrough infections; a total of 422 infections were seen amongst those with 2 + chronic conditions, with 67% (*n* = 282) being breakthrough infections.

## Discussion

Comorbid conditions are a known risk factor for severe outcomes from COVID-19 generally (21), as well as specifically for breakthrough infections (12, 22, 23). At 41% for diabetes, 73% for hypertension, 27% for COPD, and 28% for IHD, chronic disease prevalence among breakthrough cases experiencing severe outcomes in our study mirror what has been published in the literature (15, 22–25). Diabetes prevalence in hospitalized breakthrough cases has ranged from 28% to 48% (15, 22–25), hypertension 64%–71% (22–24), 24% for COPD (24), and 21% for IHD (23). From a policy level, older age groups and those with chronic conditions were prioritized in early vaccination campaigns in Manitoba; our results demonstrate that this prioritization was justified, as evidenced by the high level of chronic conditions in our breakthrough cohort, the higher likelihood of this group to be older and to have chronic conditions, compared to contemporaneous unvaccinated cases, and by the association between chronic conditions and severe outcomes demonstrated in our analyses. Not unexpectedly, a large degree of clustering of chronic conditions was observed, with approximately one out of every four of breakthrough cases having two or more chronic conditions, with the most common chronic conditions being hypertension, ischemic heart disease, and diabetes.

A combination of factors contribute to risk for and from breakthrough infections, including viral evolution, host determinants, immunity characteristics, and vaccination properties (12). Combining epidemiological data with information on immunological profiles would be a valuable future direction to further elucidate risk (26). Future work should also include examination of the determinants of breakthrough infections in the Omicron, and post-Omicron eras, as well as the mediating/moderating role that race/ethnicity may have on vulnerability to breakthrough infections and severe outcomes. As has been established in many settings, COVID-19 has disproportionately impacted Black, Indigenous and people of colour communities (27, 28); in Manitoba Indigenous communities were made a public health priority (16). Our study had a number of strengths, including the availability of population-based data sources, as well as having public health data linked to administrative healthcare databases in order to produce profiles of chronic conditions in an objective manner. Our study also had some limitations. First, we only had data on positive cases of SARS-CoV-2, and could not compare characteristics of breakthrough cases to those vaccinated and did not experience breakthrough infections. Second, the study relied on passive surveillance reporting, and thus the possibility of under-detection of cases amongst vaccinated and unvaccinated individuals exists. However, the reporting of COVID-19 cases was a priority for public health, with significant resources invested in case and contact investigations; however, stigma associated with a COVID-19 infection acting as a barrier for reporting cannot be discounted. It is important to consider as well that in a “post-Omicron” world, loss of public interest in the pandemic and the ubiquity of at-home testing, combined with less public health follow-up of cases (and their contacts) has now obscured the epidemiology of the pandemic. Third, although administrative healthcare algorithms used to define chronic conditions have been validated, the possibility for misclassification remains. However, the prevalence of key chronic conditions in our study, like diabetes and hypertension, was similar to other work. Fourth, we did not include any variables to explore the impact of race and ethnicity. Fifth, our study only captured pre-Omicron infections; however, despite the dynamic immunological landscape, in terms of the impact of previous infection from different variants on population-level immunity, in combination with timing, and dosing of COVID-19 vaccines, and the emergence of new variants, there is still strong evidence that booster shots can reduce risk from severe outcomes from COVID-19 (29). Incidentally, Although we have categorized the study period as pre vs. post-Omicron, it should be noted that a number of variants of concern circulated in Manitoba since the arrival of the wild-type virus, including the Alpha, Beta, and Gamma variants. Notably, the Delta variant arrive in Manitoba in the spring of 2021 and became the most dominant variant by the summer; this variant was responsible for Manitoba's intensive care units being overrun in the spring and summer of 2021. Finally, the association between age and breakthrough infections may be confounded by older adults being more likely to test if symptomatic.

## Conclusion

In this retrospective study, we demonstrated the high prevalence of chronic conditions and SARS-CoV-2 breakthrough infections and the risk of severe outcomes related to those infections. Future public health policy should continue to prioritize protection of those most vulnerable to COVID-19 disease.

## Data Availability

The data analyzed in this study is subject to the following licenses/restrictions: data are not available as they cannot be shared outside of the Government of Manitoba. Requests to access these datasets should be directed to Souradet Y. Shaw, souradet.shaw@umanitoba.ca.
