# Supplementary material for: The descriptive epidemiology of pre-omicron SARS-CoV-2 breakthrough infections and severe outcomes in Manitoba, Canada
Source: Front Epidemiol. 2024 Jan 12;3:1248847. doi: 10.3389/fepid.2023.1248847 (PMC10911002; doi:10.3389/fepid.2023.1248847)
Supplement: Supplementary file 1 [file Table1.docx]

**Table 1: Selected Characteristics, Breakthrough Infections in Fully Vaccinated Cases compared to Contemporaneous Matched Unvaccinated Cases in Manitoba (January 1-November 30, 2021), and Odds Ratios (ORs) and 95% Confidence Intervals (95% CI) from Conditional Logistic Regression Models (N=15,216)^*^**

|  | **Unvaccinated**  **Cases** | | **Breakthrough Cases** | | **Total** | | **ORs (95% CI)** |
| --- | --- | --- | --- | --- | --- | --- | --- |
|  | No. | % | No. | % | No. | % |  |
| Sex^*^ |  |  |  |  |  |  | -- |
| Female | 6,267 | 54.9 | 2,089 | 54.9 | 8,356 | 54.9 |  |
| Male | 5,145 | 45.1 | 1,715 | 45.1 | 6,860 | 45.1 |  |
| Age group |  |  |  |  |  |  |  |
| 18-29 | 1,920 | 16.8 | 640 | 16.8 | 2,560 | 16.8 | *--* |
| 30-39 | 2,304 | 20.2 | 768 | 20.2 | 3,072 | 20.2 |  |
| 40-49 | 2,208 | 19.3 | 736 | 19.3 | 2,944 | 19.3 |  |
| 50-59 | 1,674 | 14.7 | 558 | 14.7 | 2,232 | 14.7 |  |
| 60-69 | 1,494 | 13.1 | 498 | 13.1 | 1,992 | 13.1 |  |
| 70+ | 1,812 | 15.9 | 604 | 15.9 | 2,416 | 15.9 |  |
| Regional Health Authority |  |  |  |  |  |  |  |
| Interlake-Eastern | 886 | 7.8 | 338 | 8.9 | 1,224 | 8.0 | 0.56 (0.48-0.66) |
| Northern | 588 | 5.2 | 620 | 16.3 | 1,208 | 7.9 | 1.60 (1.39-1.84) |
| Prairie Mountain | 1,313 | 11.5 | 504 | 13.2 | 1,817 | 11.9 | 0.49 (0.43-0.56) |
| Southern | 6,404 | 56.1 | 923 | 24.3 | 7,327 | 48.2 | 0.19 (0.17-0.21) |
| Winnipeg | 2,221 | 19.5 | 1,419 | 37.3 | 3,640 | 23.9 | *Ref* |
| Month (epi-date)^*^ |  |  |  |  |  |  | -- |
| Feb | 2 | 0.0 | 1 | 0.0 | 3 | 0.0 |  |
| Mar | 32 | 0.3 | 9 | 0.2 | 41 | 0.3 |  |
| Apr | 96 | 0.8 | 31 | 0.8 | 127 | 0.8 |  |
| May | 390 | 3.4 | 127 | 3.3 | 517 | 3.4 |  |
| Jun | 206 | 1.8 | 64 | 1.7 | 270 | 1.8 |  |
| Jul | 308 | 2.7 | 99 | 2.6 | 407 | 2.7 |  |
| Aug | 752 | 6.6 | 277 | 7.3 | 1,029 | 6.8 |  |
| Sep | 1,636 | 14.3 | 518 | 13.6 | 2,154 | 14.2 |  |
| Oct | 2,957 | 25.9 | 917 | 24.1 | 3,874 | 25.5 |  |
| Nov | 5,033 | 44.1 | 1,761 | 46.3 | 6,794 | 44.7 |  |
| Chronic conditions |  |  |  |  |  |  |  |
| 0 | 7,060 | 61.9 | 2,039 | 53.6 | 9,099 | 59.8 | *Ref* |
| 1 | 2,364 | 20.7 | 862 | 22.7 | 3,226 | 21.2 | 1.37 (1.24-1.51) |
| 2 | 1,499 | 13.1 | 665 | 17.5 | 2,164 | 14.2 | 1.89 (1.67-2.13) |
| 3 | 372 | 3.3 | 191 | 5.0 | 563 | 3.7 | 2.37 (1.94-2.90) |
| 4+ | 117 | 1.0 | 47 | 1.2 | 164 | 1.1 | 1.94 (1.36-2.77) |
| Stroke |  |  |  |  |  |  |  |
| No | 11,055 | 96.9 | 3,670 | 96.5 | 14,725 | 96.8 | *Ref* |
| Yes | 357 | 3.1 | 134 | 3.5 | 491 | 3.2 | 1.14 (0.92-1.40) |
| Heart failure |  |  |  |  |  |  |  |
| No | 11,124 | 97.5 | 3,643 | 95.8 | 14,767 | 97.0 | *Ref* |
| Yes | 288 | 2.5 | 161 | 4.2 | 449 | 3.0 | 1.81 (1.47-2.23) |
| Acute Myocardial Infarction |  |  |  |  |  |  |  |
| No | 11,152 | 97.7 | 3,702 | 97.3 | 14,854 | 97.6 | *Ref* |
| Yes | 260 | 2.3 | 102 | 2.7 | 362 | 2.4 | 1.19 (0.94-1.52) |
| Ischemic Heart Disease |  |  |  |  |  |  |  |
| No | 11,152 | 97.7 | 3,702 | 97.3 | 14,854 | 97.6 | *Ref* |
| Yes | 260 | 2.3 | 102 | 2.7 | 362 | 2.4 | 1.49 (1.28-1.75) |
| Diabetes |  |  |  |  |  |  |  |
| No | 10,125 | 88.7 | 3,153 | 82.9 | 13,278 | 87.3 | *Ref* |
| Yes | 1,287 | 11.3 | 651 | 17.1 | 1,938 | 12.7 | 1.73 (1.55-1.93) |
| Hypertension |  |  |  |  |  |  |  |
| No | 8,540 | 74.8 | 2,652 | 69.7 | 11,192 | 73.6 | *Ref* |
| Yes | 2,872 | 25.2 | 1,152 | 30.3 | 4,024 | 26.4 | 1.47 (1.33-1.63) |
| Chronic Obstructive Pulmonary Disease |  |  |  |  |  |  |  |
| No | 10,682 | 93.6 | 3,486 | 91.6 | 14,168 | 93.1 | *Ref* |
| Yes | 730 | 6.4 | 318 | 8.4 | 1,048 | 6.9 | 1.40 (1.20-1.62) |
| Asthma |  |  |  |  |  |  |  |
| No | 10,007 | 87.7 | 3,263 | 85.8 | 13,270 | 87.2 | *Ref* |
| Yes | 1,405 | 12.3 | 541 | 14.2 | 1,946 | 12.8 | 1.18 (1.06-1.32) |
| Parkinson’s |  |  |  |  |  |  |  |
| No | 11,388 | 99.8 | 3,793 | 99.7 | 15,181 | 99.8 | *Ref* |
| Yes | 24 | 0.2 | 11 | 0.3 | 35 | 0.2 | 1.38 (0.67-2.81) |
| Multiple Sclerosis |  |  |  |  |  |  |  |
| No | 11,382 | 99.7 | 3,798 | 99.8 | 15,180 | 99.8 | *Ref* |
| Yes | 30 | 0.3 | 6 | 0.2 | 36 | 0.2 | 0.60 (0.25-1.44) |
| Epilepsy |  |  |  |  |  |  |  |
| No | 11,355 | 99.5 | 3,761 | 98.9 | 15,116 | 99.3 | *Ref* |
| Yes | 57 | 0.5 | 43 | 1.1 | 100 | 0.7 | 2.29 (1.54-3.42) |
| Osteoarthritis |  |  |  |  |  |  |  |
| No | 11,188 | 98.0 | 3,667 | 96.4 | 14,855 | 97.6 | *Ref* |
| Yes | 224 | 2.0 | 137 | 3.6 | 361 | 2.4 | 2.13 (1.67-2.72) |

*matched on age group, sex, and epidemiological date (1:4 ratio)
