# Supplementary material for: The descriptive epidemiology of pre-omicron SARS-CoV-2 breakthrough infections and severe outcomes in Manitoba, Canada
Source: Front Epidemiol. 2024 Jan 12;3:1248847. doi: 10.3389/fepid.2023.1248847 (PMC10911002; doi:10.3389/fepid.2023.1248847)
Supplement: Supplementary file 2 [file Table2.docx]

**Table 2: Unadjusted and Adjusted Hazard Ratios (U/AHRs) and 95% Confidence Intervals (95%CI) from Cox Regression Models examining Determinants of Hospitalizations/ICU admissions amongst Breakthrough Infection Cases in Manitoba, January 1-November 30, 2021 (N=3,706)**

|  | | **Hospitalized/ICU** | |  |  |
| --- | --- | --- | --- | --- | --- |
| **Variables** | | **No**  **No. (%)** | **Yes**  **No. (%)** | **AHR** | **95% CI** |
|  | | 3,540 (96.2) | 166 (4.5) |  |  |
| Sex | Female | 1,945 (54.9) | 83 (50.0) | *Ref* | -- |
|  | Male | 1,595 (45.1) | 83 (50.0) | 1.26 | [0.90,1.77] |
| Age group | 18-29 | 630 (17.8) | 6 (3.6) | *Ref* | -- |
|  | 30-39 | 757 (21.4) | 8 (4.8) | 1.04 | [0.28,3.87] |
|  | 40-49 | 718 (20.3) | 15 (9.0) | 2.12 | [0.67,6.70] |
|  | 50-59 | 533 (15.1) | 14 (8.4) | 1.93 | [0.58,6.41] |
|  | 60-69 | 445 (12.6) | 37 (22.3) | 5.80^**^ | [1.91,17.59] |
|  | 70+ | 457 (12.9) | 86 (51.8) | 13.3^***^ | [4.45,39.76] |
|  | Mean (years), Median (IQR) | 47.1,  44 (33-60) | 71.0,  70 (59-83) |  |  |
| 1^st^ & 2^nd^ Dose | <30 days | 935 (26.4) | 45 (27.1) | *Ref* | -- |
| Interval | 30-59 days | 1,797 (50.8) | 78 (47.0) | 1.33 | [0.86,2.06] |
|  | 60+ days | 808 (22.8) | 43 (25.9) | 0.68 | [0.42,1.10] |
| Regional Health Authority | Interlake-Eastern | 312 (8.8) | 16 (9.6) | 0.57 | [0.31,1.02] |
|  | Northern | 588 (16.6) | 24 (14.5) | 0.71 | [0.41,1.22] |
|  | Prairie Mountain | 451 (12.7) | 27 (16.3) | 0.73 | [0.44,1.23] |
|  | Southern | 862 (24.4) | 34 (20.5) | 0.45^**^ | [0.27,0.74] |
|  | Winnipeg | 1,327 (37.5) | 65 (39.2) | *Ref* | -- |
| Chronic Conditions | 0 | 2,001 (56.5) | 24 (14.5) | *Ref* | -- |
|  | 1 | 817 (23.1) | 31 (18.7) | 1.57 | [0.83,2.97] |
|  | 2+ | 722 (20.4) | 111 (66.9) | 3.55^***^ | [1.98,6.38] |
|  | Median (IQR) | 0 (0-1) | 2 (1-4) |  |  |

^*^ *p* < 0.05, ^**^ *p* < 0.01, ^***^ *p* < 0.001
