# Supplementary material for: The descriptive epidemiology of pre-omicron SARS-CoV-2 breakthrough infections and severe outcomes in Manitoba, Canada
Source: Front Epidemiol. 2024 Jan 12;3:1248847. doi: 10.3389/fepid.2023.1248847 (PMC10911002; doi:10.3389/fepid.2023.1248847)
Supplement: Supplementary file 3 [file Table3.docx]

**Table 3: Adjusted Hazard Ratios (AHRs^*^) and 95% Confidence Intervals (95% CI) from Separate Cox Regression Models, Association between Specific Chronic Conditions and Hospitalizations/ICU Admissions among Breakthrough Infection Cases in Manitoba, January 1-November 30, 2021 (N=3,706)**

|  |  | **Prevalence**  **(%)** | |
| --- | --- | --- | --- |
| **Condition** | **AHR (95% CI) ^a^** | **Non-Hospitalized** | **Hospitalized** |
| Stroke | 1.19 (0.69-2.08) | 3.0 | 11.2 |
| Heart Failure | **3.25 (2.15-4.93)** | 2.89 | 26.9 |
| Myocardial Infarction | 1.66 (0.94-2.94) | 2.2 | 10.4 |
| Ischemic Heart Disease | **1.63 (1.09-2.44)** | 6.2 | 28.4 |
| Diabetes | **1.99 (1.38-2.87)** | 15.0 | 44.0 |
| Hypertension | **2.41 (1.51-3.84)** | 27.0 | 74.6 |
| COPD | **1.74 (1.17-2.58)** | 6.9 | 29.1 |
| Asthma | 1.35 (0.87-2.11) | 14.0 | 17.9 |
| Parkinson’s Disease | 2.41 (0.59-9.88) | 0.2 | 1.5 |
| Multiple Sclerosis | -- | -- | -- |
| Epilepsy | 1.36 (0.43-4.28) | 1.0 | 2.2 |
| Osteoarthritis | 1.58 (0.94-2.66) | 2.8 | 14.9 |
| Any chronic condition | **2.81 (1.55-5.08)** | 45.8 | 88.1 |

*adjusted for age group, sex, dose interval, & regional health authority
